# Adoption of the cardiopulmonary exercise test in the exercise ability and cardiopulmonary function rehabilitation of coronary artery disease (CAD) patients

**DOI:** 10.1186/s12872-024-03958-0

**Published:** 2024-06-21

**Authors:** Lingling Wang, Fan Mei, Mengyi Min, Xiuyan He, Lili Luo, Youxia Ma

**Affiliations:** 1 Department of Cardiology,Xiangyang No.1, People’s Hospital, Hubei University of Medicine, Xiangyang, Hubei 441000 China; 2 Nursing Department, Grade 7, Health Center of Jimo District, Qingdao, Shandong 266200 China; 3 Nursing Department, Duanbolan Health Center of Jimo District, Qingdao, Shandong 266200 China; 4 Nursing Department, Shandong First Medical University, Taian, Shandong 271000 China; 5 Pulmonary and Critical Care Medicine Nursing Care, Laishan Hosital, Yantaishan Hospital, Yantai, 264000 China

**Keywords:** Rehabilitation training, Cardiopulmonary, Coronary artery disease, Blood lipid levels

## Abstract

**Background:**

This study aimed to explore the application of cardiopulmonary exercise testing in coronary artery disease (CAD) patients, evaluate its impact on exercise ability and cardiopulmonary function in patients with coronary heart disease (CHD), and promote the application of cardiopulmonary exercise testing in CAD management.

**Methods:**

Fifty CHD patients after percutaneous coronary intervention (PCI) were recruited and randomly enrolled into the control (Ctrl) group and intervention (Int) group. Routine health education and health education combined with RT training were carried out for the two groups. Blood lipid levels and lung function were compared between the two groups after intervention. Cardiac function was evaluated by Doppler ultrasonography, and cardiopulmonary fitness and exercise ability were evaluated by a cardiopulmonary exercise test (CPET). The self-rating anxiety scale (SAS) and self-rating depression scale (SDS) were employed to evaluate negative emotions. The 36-item short-form (SF-36) was adopted to evaluate quality of life.

**Result:**

: Compared with those in the Ctrl group, the levels of serum total cholesterol (TC), triglycerides (TGs), high-density lipoprotein (HDL), and low-density lipoprotein (LDL) decreased in the Int group, while the levels of high-density lipoprotein increased (*P* < 0.05). The quantitative load results showed that compared with the Ctrl group, the heart rate (HR) and self-perceived fatigue degree of the Int group decreased, and the ST segment increased (*P* < 0.05). Compared with the Ctrl group, the left ventricular ejection fraction (LVEF), forced expiratory volume at 1 s (FEV_1_), ratio of forced expiratory volume to forced vital volume (FEV_1_/FVC%), and maximum chase volume (MVV) increased in the Int group, while the left ventricular end diastolic diameter and left ventricular end contractile diameter decreased (*P* < 0.05). The results of the CPET showed that compared with the Ctrl group, minute ventilation/carbon dioxide production slope, VE/VCO_2 − Peak_, anaerobic threshold (AT), peak oxygen pulse (VO_2_/HR _peak_), oxygen uptake efficiency platform (OUEP), increasing power exercise time (IPEt), HR recovery 1 min after exercise, peak load power (Watt _peak_), and value metabolic equivalent (Watt _peak_) increased in the Int group (*P* < 0.05). Compared with the Ctrl group, the SAS and SDS scores in the Int group decreased (*P* < 0.05). The results of the quality of life evaluation showed that compared with the Ctrl group, the score of the SF-36 dimensions increased in the Int group (*P* < 0.05).

**Conclusion:**

RT training can reduce postoperative blood lipid and quantitative load levels in CAD patients and improve adverse mood. Furthermore, it can improve patients’ cardiopulmonary function, cardiopulmonary fitness, exercise ability, and quality of life.

## Background

Coronary artery disease (CAD) is a prevalent cardiovascular ailment, also known as coronary heart disease (CHD) [1]. In recent years, the global incidence of CAD has exhibited an increasing trend, rising from 186 million individuals in 2017 to 208 million in 2021, with a compounded annual growth rate of 2.8%. Preliminary estimates suggest that by 2022, the worldwide population of CAD patients has reached approximately 213 million [2]. Percutaneous coronary intervention (PCI) serves as the principal modality for CAD treatment, facilitating the enhancement of myocardial blood supply, the reconstruction of ischemic myocardium, and the reduction of mortality risk [3]. Nevertheless, PCI does not represent the ultimate endpoint of CAD patient management. In addition to the continuation of conventional pharmacological treatments postprocedure, cardiac rehabilitation assumes critical significance [4]. Clinical guidelines advocate that cardiac rehabilitation, through personalized rehabilitation programs, aims to enhance the cardiovascular health of patients, improving their physiological, psychological, social, occupational, and emotional well-being [5]. Research indicates that moderate exercise is instrumental in ensuring the safety of CAD patients and reducing complications stemming from prolonged bed rest [6]. Cardiac rehabilitation training stimulates physiological adaptation by increasing peak oxygen uptake, enhancing physical fitness and quality of life in cardiovascular disease patients, and lowering all-cause mortality and hospital readmission rates [7, 8].

Cardiac exercise training (ET) represents a targeted physical exercise program designed to enhance cardiac health, improve aerobic capacity, and increase exercise tolerance [9]. Typically, supervised by a professional medical team or trainers, this training is specifically tailored to individuals afflicted with heart disease and other cardiovascular risk groups. Its purpose is to assist them in ameliorating their cardiovascular health status, alleviating symptoms, enhancing their quality of life, and reducing the risk of complications [10]. Nonetheless, in the current context of China, there are certain limitations pertaining to the promotion and application of cardiac rehabilitation, particularly cardiac ET for CAD patients. Part of the reason for this situation is the insufficient awareness of the risks associated with exercise rehabilitation and a relatively weak understanding of the importance of rehabilitation among the patient population. Consequently, it is of utmost significance to actively promote the implementation of cardiac ET for CAD patients. Left ventricular ejection fraction (LVEF) is a measurement indicator of heart morphology and structure and is often used in prognosis prediction and evaluation of patients [11]. Compared to the LVEF, the cardiopulmonary exercise test (CPET) is completed when the lung capacity, lung dispersion function, and other lung functions are detected under the resting state. Subsequently, metabolic indicators such as ECG, blood oxygen level, CO_2_ emission, and other indicators of symptom limitation in the resting state were continuously monitored [12]. CPET is currently the only method that can integrate objective measurement and evaluation of cardiopulmonary metabolic function and can also comprehensively evaluate the overall function of the collective [13]. Maximal oxygen uptake measured by CPET has become the gold standard of aerobic exercise tolerance and the anaerobic threshold [14].

Therefore, we hypothesized that the combined application of ET and pharmaceutical treatment can significantly enhance the cardiopulmonary function of CAD patients, improve their exercise capacity, and have a positive impact on their rehabilitation. Accordingly, the primary objective of this study was to explore the effects of standard pharmacological treatment in conjunction with ET on the cardiopulmonary function and exercise capacity of CAD patients following PCI, employing techniques such as cardiopulmonary exercise testing (CPET) and other relevant assessments. Additionally, the effectiveness of CPET in objectively measuring and evaluating the cardiopulmonary metabolic function of CAD patients was also assessed, providing deeper insights into their physiological status. This, in turn, will better inform the development of rehabilitation programs and offer novel pathways for the rehabilitation of CAD patients.

## Materials and methods

### Study flowchart

#### The flowchart for the current study is illustrated in Fig. 1

**Fig. 1:**
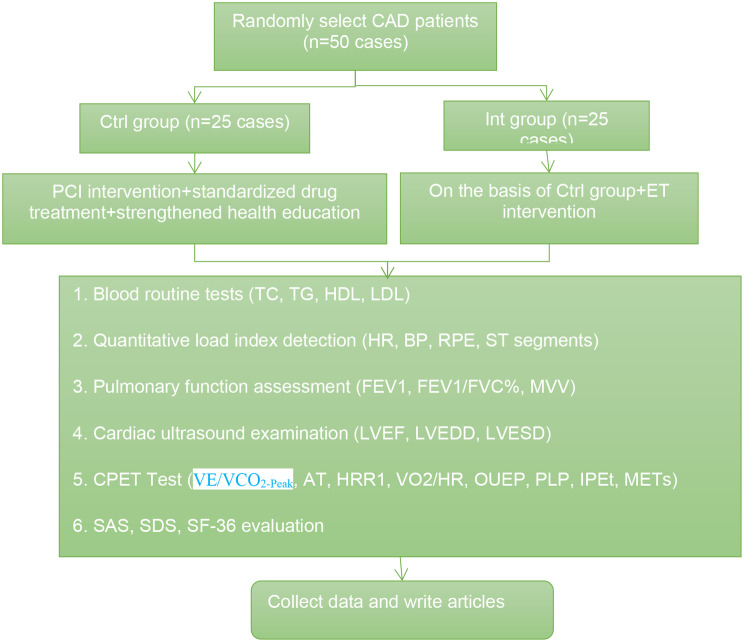
Study flowchart

### General Information

For this study, a total of 50 CAD patients who underwent PCI intervention at the Department of Cardiology and Cardiac Rehabilitation Outpatient Clinic of the First People’s Hospital of Xiangyang from June 2020 to December 2021 were randomly selected. Patients were allocated into two groups using a random number table: the Ctrl group (comprising 25 patients receiving combined standard pharmacological treatment and enhanced health education) and the Int group (consisting of 25 patients who received ET intervention in addition to the Ctrl group’s treatment). The study aimed to analyze the impact of these two treatment approaches on the rehabilitation outcomes of CAD patients. The research adhered to the principles outlined in the *Helsinki Declaration*. Furthermore, in accordance with applicable legal regulations and medical ethical guidelines, all subjects participating in the study were adequately informed about the potential risks, benefits, and psychological stress associated with physical exercise, and they provided informed consent. The specific inclusion and exclusion criteria for the study participants are as follows:

Inclusion criteria: (i) patients meeting the diagnostic criteria for CAD; (ii) patients who met the exercise experiment and training criteria published by the American Heart Association in 2013; (iii) patients with evident ischemic changes on resting ECG and/or myocardial ischemia on CPET at attack; (iv) patients who were treated with coronary artery stenting; (v) patients who had no continuous exercise habit in the past exercised less than twice per week and each exercise time was less than 15 min; (vi) the *New York Heart Association* cardiac function grading was grade II to III; and (vii) the patient was stable and able to receive exercise therapy.

Exclusion criteria: (i) patients with acute myocardial infarction occurring within the last 30 days; (ii) patients with chronic obstructive pulmonary disease, pulmonary embolism, stroke, and other diseases that can affect lung function; (iii) patients with unstable angina pectoris, or grade IV angina pectoris; (iv) patients with severe arrhythmia, cardiac insufficiency, or aortic dissection/stenosis; (v) patients with severe liver and kidney dysfunction; (vi) those who suffered from language communication disorder, hearing disorder, and mental dysfunction affecting communication; (vii) patients with infectious diseases, malignant tumors, or diseases of the blood system; and (viii) patients with rheumatism or motor diseases affecting training.

#### Therapy methods

Interventional treatment was performed. All patients underwent PCI treatment. During the surgical procedure, patients were placed in a supine position, and local anesthesia was administered at the right wrist using 1% lidocaine (B. Braun Melsungen AG, Germany). Subsequently, the Seldinger technique was employed to puncture the right radial artery, and a 6 F arterial sheath introducer (Terumo Corporation, Japan) was inserted. Following sheath placement, an appropriate dose of heparin (Pfizer Pharmaceuticals, USA) and nitroglycerin (AstraZeneca, UK) was administered, and multiposition coronary angiography was performed using a 5 F multifunctional catheter (Cordis Corporation, USA) to assess the condition of the coronary arteries. Once the degree of stenosis was confirmed (stenosis > 80%, with residual stenosis < 20% poststent deployment), PCI was performed, and the expansion and adhesion of the stent were assessed postdeployment. For patients who received thrombolytic therapy in the target vessel due to myocardial infarction, a clinical trial-based flow assessment was utilized for evaluation.

Patients in the Ctrl group received standardized pharmacological treatment and enhanced health education. Health education related to CAD was provided to the patients, including dietary habits: patients were informed about the impact of diet on CAD and educated to choose healthy foods, including a low-fat, low-cholesterol diet rich in vegetables and fruits, and to reduce salt intake. Furthermore, they were instructed on how to manage their diet to maintain a healthy weight; healthy lifestyle: promoting a healthy lifestyle is crucial for CAD patients. Patients were encouraged to quit smoking, limit alcohol intake, increase physical activity, control chronic conditions such as hypertension and diabetes, and undergo regular check-ups; psychological support: CAD can have a negative impact on patients’ psychological well-being. Therefore, providing psychological support and education to help patients cope with anxiety, depression, and psychological stress is essential. This included psychological therapy, relaxation techniques, and introductions to support groups; survival education for cardiovascular emergencies: patients were educated on how to respond to potential cardiac emergencies, including angina and heart attacks. They needed to understand how to use emergency medications, make emergency calls, and take immediate action when symptoms occurred.

In the Int group, in addition to the treatment methods applied in the Ctrl group, patients received ET interventions. The following is a detailed description of the ET intervention: warm-up exercises: before commencing the formal ET, patients engaged in 5 to 10 min of warm-up exercises. The purpose of this warm-up phase was to prepare the body for higher-intensity exercise and reduce the risk of injury. Warm-up exercises primarily included low-intensity aerobic activities or low-intensity stretching exercises. Formal exercise: formal ET training encompassed fixed treadmill running, treadmill exercises, or upper body fluid resistance training (NordicTrack T Series, USA). These exercises were predominantly aerobic in nature and aimed at improving cardiopulmonary health, enhancing muscular strength, and promoting overall well-being. Exercise intensity assessment: to ensure patient safety, exercise intensity needed assessment. The Berg Balance Scale is a commonly used tool for assessing the risk of falls in elderly individuals. A score within the range of 13 to 16 on this scale was necessary to ensure that patients had sufficient exercise capacity to undertake formal exercise. Training frequency and duration: the duration of each formal aerobic ET session ranged from 30 to 60 min. The training frequency was set at 3 to 4 sessions per week, with a 1-day interval between each training session. This training schedule was designed to promote improvements in cardiopulmonary health and physical adaptability. Electrocardiogram (ECG) monitoring: To monitor the cardiac status of patients during exercise, a remote ECG telemetry system (Siemens Remote Service system, Siemens Healthineers AG, Germany) was employed. This allowed for the timely detection of ECG abnormalities to ensure patient safety. Stop criteria: If patients experienced progressive chest pain, pallor, dizziness, fatigue, shortness of breath, or discomfort during exercise, they were instructed to immediately cease the activity. These symptoms could be indicative of cardiovascular issues. Following exercise cessation, the physician might need to administer medications such as nitroglycerin based on the patient’s condition. The specific parameters of ET, including frequency, intensity, time, and type (FITT), are detailed in Table 1.

**Table 1:** Specific indicators of FITT parameters

FITT parameter	Frequency	Intensity (Borg Scale)	Time	Type
Warm-up	3–4 times per week	——	5–10 min/time	Aerobic exercise
Formal sports	3–4 times per week	13 ~ 16 points	30 ~ 60 min/time	Aerobic exercise

#### Routine blood indicators

In this study, the Hitachi 7170 A Automatic Analyzer (manufactured by Hitachi, Japan) was employed to measure the levels of total cholesterol (TC), triglycerides (TG), high-density lipoprotein (HDL), and low-density lipoprotein (LDL). This analysis served to assess cardiovascular health and risk factors.

#### Quantitative load index

Heart rate (HR), blood pressure (BP), rating of perceived exertion (RPE), and ST segment were measured using the Cortex Biophysik Metamax II Gas Analysis System (Cortex Biophysik GmbH, Germany).

#### Pulmonary function evaluation

Static lung function was assessed using the MINAO AS-507 Spirometer (MINATO, Japan). The primary parameters measured included forced expiratory volume in 1 s (FEV1), the ratio of forced expiratory volume in 1 s to forced vital capacity (FEV1/FVC%), and maximum voluntary ventilation (MVV).

#### Cardiac ultrasound examination

Cardiac function was assessed in the morning from 9 to 10 using a cardiac color Doppler ultrasound diagnostic system (General Electric Healthcare, USA). This evaluation encompassed the measurement of key parameters, including left ventricular ejection fraction (LVEF), left ventricular end-diastolic diameter (LVEDD), and left ventricular end-systolic diameter (LVESD). The specific procedure was as follows: patients were instructed to lie supine on the examination bed, and the ultrasound probe was set at a frequency of 8.2 Hz. Subsequently, the gel-coated ultrasound probe was placed on the patient’s chest, covering areas including below the sternum and to the left of the sternum, to acquire the LVEF, LVEDD, and LVESD parameters of the heart.

#### CPET test

In this study, the assessment of patients’ cardiopulmonary function, respiratory system efficiency, and exercise capacity was conducted before and after the intervention using the PFTErgo V10.0 A cardiopulmonary exercise testing (CPET) system from COSMED (Italy). The CPET testing in this study utilized a stationary bike protocol with a progressive workload increase every minute, allowing patients to reach their maximum achievable load within 8–12 min. After increasing the workload on the stationary bike, patients continued exercising on a treadmill and were subsequently monitored after cessation of exercise. Clinically, the peak values between 8 and 12 min are commonly used to assess patient rehabilitation progress. Data from the CPET examination were determined based on standardized principles. The primary parameters included the minute ventilation-to-carbon dioxide production slope (VE/VCO_2 − Peak_), anaerobic threshold (AT), peak oxygen pulse (VO_2_/HR _Peak_), 1-minute postexercise heart rate recovery (HRR1), oxygen uptake efficiency plateau (OUEP), peak load power (PLP), increased power exercise time (IPEt), and peak metabolic equivalents (METs).

#### Evaluation of anxiety and depression

The Self-Rating Anxiety Scale (SAS) [15] and Self-Rating Depression Scale (SDS) [16] were adopted to evaluate anxiety and depression, respectively. The SAS and SDS contain 20 items, and each item is divided into 4 levels. When the score is less than 50, it indicates that the patient’s mood is normal, and there is no obvious anxiety or depression. Scores of more than 50 indicate significant anxiety or depression. The higher the SAS and SDS scores were, the more serious the anxiety or depression.

#### 36-item short-form (SF-36) health status assessment

Patients’ health status was evaluated using the SF-36 scale [17], which includes 36 items and 8 dimensions. They are physiological function (PF), physiological function (RP), physical pain (BP), general health status (GH), energy (VT), social function (SF), emotional function (RE), and mental health (MH). The higher the SF-36 score is, the better the patient’s health.

#### Outcome measures

The primary outcome measures in this study encompassed pulmonary function assessment parameters (FEV1, FEV1/FVC, MVV), cardiac function indicators (LVEF, LVEDD, LVESD), and CPET test results (peak VO_2_, AT, VO_2_/HR _peak_, OUEP, PLP, IPEt, and METs).

The secondary outcome measures in this study comprised lipid profile results (TC, TG, HDL, LDL), quantitative load index results (HR, BP, RPE, ST segment), SAS, SDS, and SF-36 assessment outcomes.

### Statistical analysis

SPSS 19.0 was employed. The mean ± SD was used for measurement data consistent with a normal distribution, and an independent sample *T* test was adopted for analysis of comparisons between the two groups. The frequency (percentage) of counting data was expressed, and the chi-square test was performed to analyze the comparison between the two groups. Pearson correlation analysis was implemented to analyze the correlation between CPET and LVEF. *P* < 0.05 indicated a statistically significant difference.

## Results

### Comparison of general data of CAD patients

The differences in general data between groups were compared. There were no marked differences in average age, sex ratio, smoking history, or BMI between groups (*P* > 0.05) (Table 2).

**Table 2:** Comparison of clinical data between groups

Data	Control group (*n* = 25)	Intervention group (*n* = 25)	Statistics	*P*
Age (years old)	57.81 ± 4.47	57.53 ± 4.31	*t* = -0.153	0.886
Sex [n (%)]			*χ*^2^ = 0.197	0.793
Meale	11 (44.0)	12 (48.0)		
Female	14 (56.0)	13 (52.0)		
Smoking [n (%)]			*χ*^2^ = 0.204	0.698
Yes	9 (36.0)	8 (32.0)		
No	16 (64.0)	17 (68.0)		
BMI (kg/m^2^)	24.89 ± 2.31	24.95 ± 1.55	*t* = 0.089	0.917

**Fig. 2:**
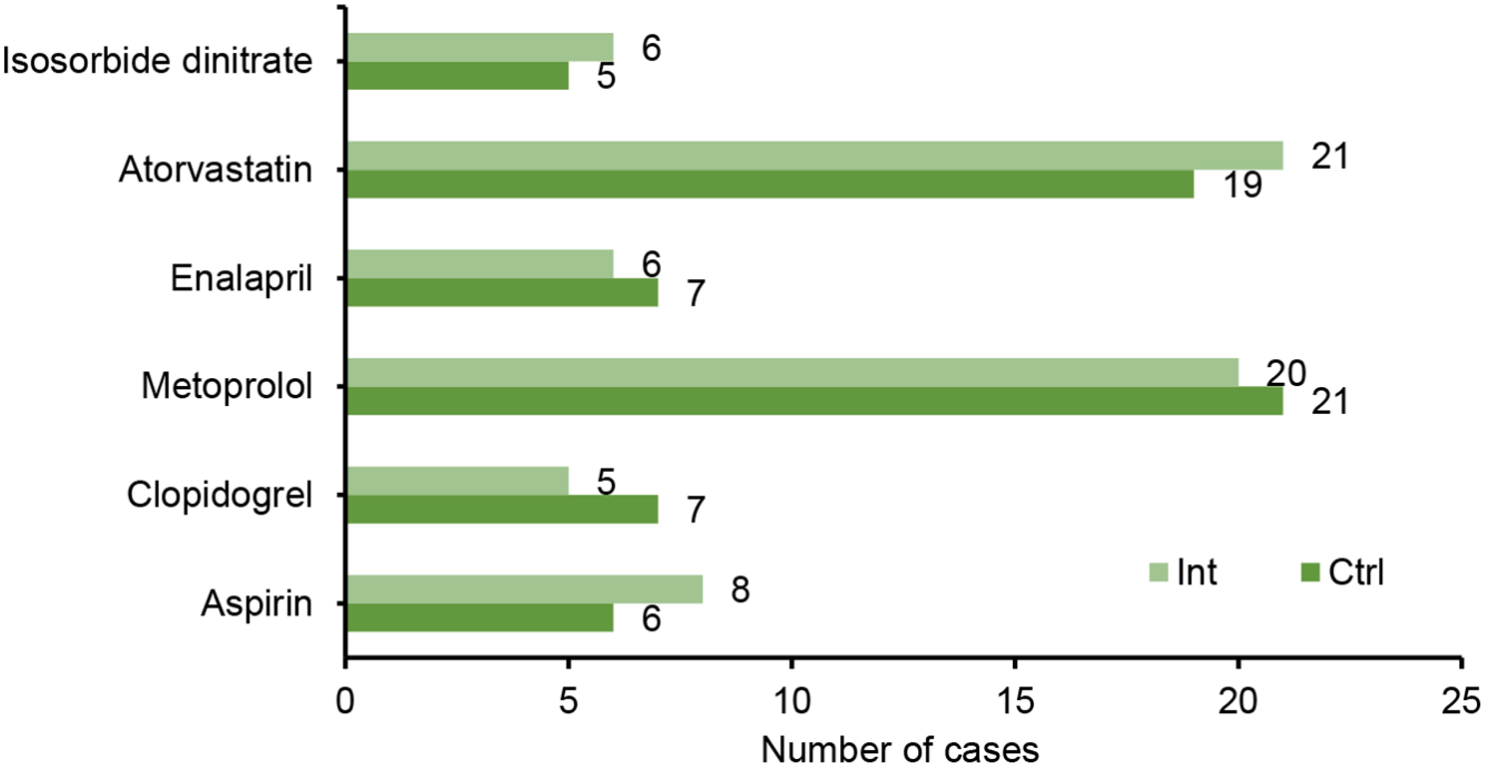
Drug use in the Ctrl group and Int group

Analysis of drug use in the Ctrl group and Int group showed that there was no considerable difference in the proportion of aspirin, clopidogrel, metoprolol, enalapril, atorvastatin, and isosorbide nitrate between groups (*P* > 0.05) (Fig. 2)

### Effect of RT on lipid levels of CAD patients

**Fig. 3:**
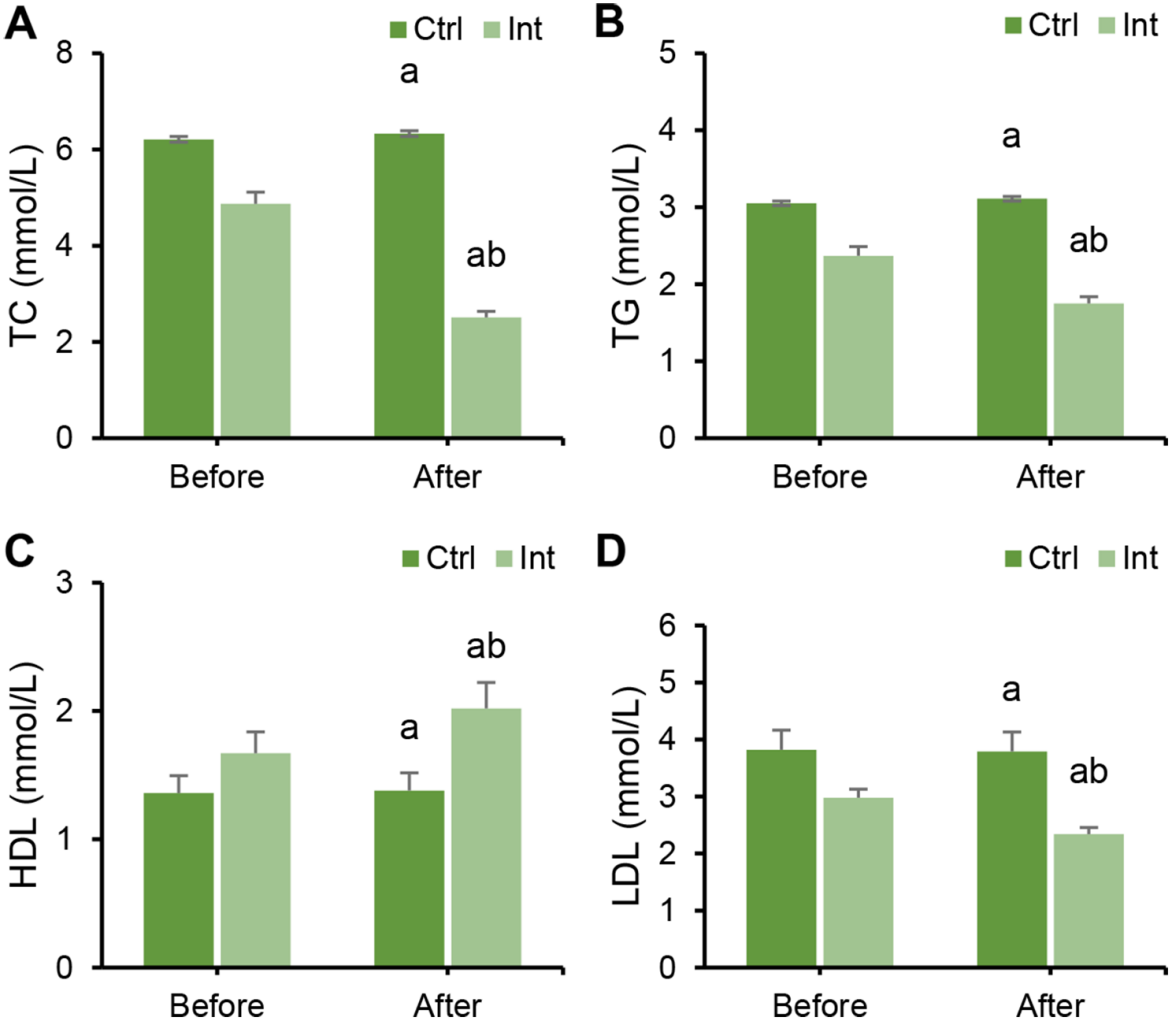
Changes in serum lipid levels in the Ctrl group and Int group before and after intervention. (**A**) TC; (**B**) TG; (**C**) HDL; (**D**) LDL; ^a^*P*<0.05 vs. that before intervention; ^b^*P*<0.05 vs. that of the postintervention Ctrl group

The levels of TC, TG, HDL, and LDL were compared between groups before and after intervention. Before intervention, there were no marked differences in TC, TG, HDL, and LDL levels between groups (*P* > 0.05). After intervention, TC, TG, and LDL levels in the Ctrl group and Int group were greatly inferior to those before intervention, and the HDL level was drastically superior to that before intervention (*P* < 0.05). After intervention, TC, TG, and LDL levels in the Int group were substantially inferior to those in the Ctrl group, while HDL levels were evidently superior to those in the Ctrl group (*P* < 0.05) (Fig. 3).

### Effect of RT on the quantitative load of CAD patients

**Fig. 4:**
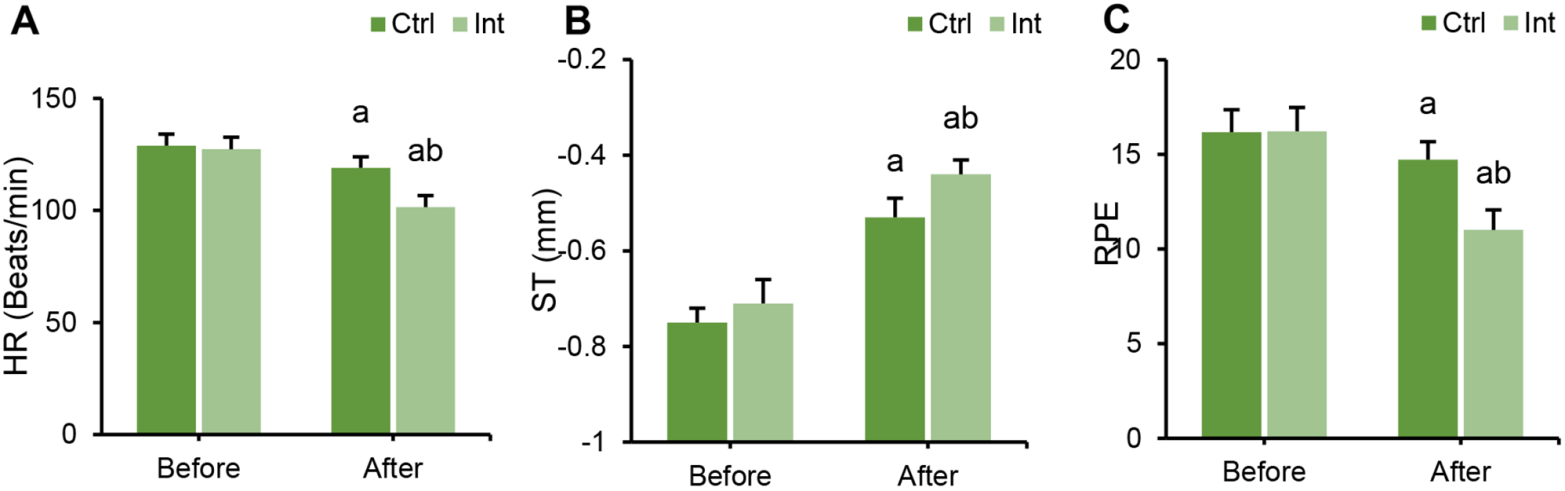
Quantitative load changes in the Ctrl group and Int group before and after intervention. (**A**) HR; (**B**) ST segment indicators; (**C**) RPE indicators; ^a^*P*<0.05 vs. that before intervention; ^b^*P*<0.05 vs. that of the postintervention Ctrl group

The differences in HR, ST segment, and RPE levels between groups before and after intervention were compared. Before intervention, there were no marked differences in HR, ST segment, or RPE levels between groups (*P* > 0.05). After intervention, the HR and RPE levels in the Ctrl group and Int group were obviously lower than those before intervention, and the ST segment level was drastically superior to that before intervention (*P* < 0.05). After intervention, HR and RPE levels in the Int group were dramatically inferior to those in the Ctrl group, and ST segment levels were evidently higher versus the Ctrl group (*P* < 0.05) (Fig. 4).

### Effect of RT on the cardiac function of patients with CAD

**Fig. 5:**
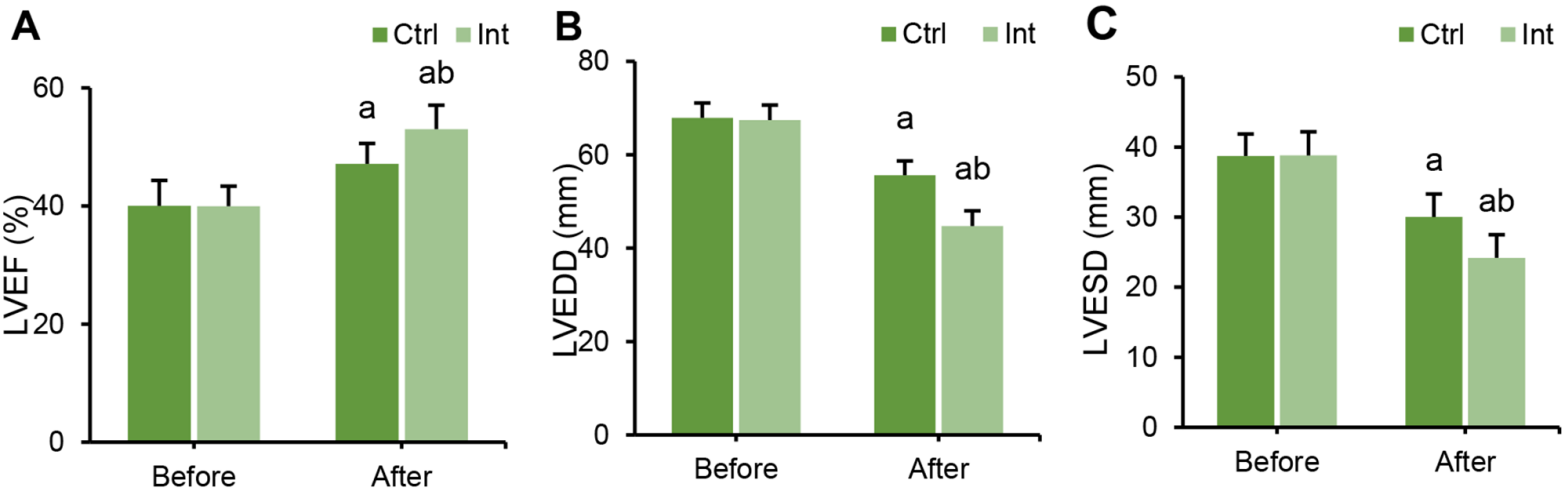
Changes in cardiac function in the Ctrl group and Int group before and after intervention. (**A**) LVEF; (**B**) LVEDD; (**C**) LVESD; ^a^*P*<0.05 vs. that before intervention; ^b^*P*<0.05 vs. that of the postintervention Ctrl group

Color Doppler ultrasound was used to evaluate the differences in cardiac function indexes LVEF, LVEDD, and LVESD between groups before and after intervention. Before intervention, there was no considerable difference in LVEF, LVEDD, or LVESD levels between groups (*P* > 0.05). After intervention, LVEDD and LVESD levels in the Ctrl group and Int group were much higher than those before intervention, and LVEF levels were drastically superior to those before intervention (*P* < 0.05). After intervention, LVEDD and LVESD levels in the Int group were much higher than those in the Ctrl group, and LVEF levels were evidently superior to those in the Ctrl group (*P* < 0.05) (Fig. 5).

### Effect of RT on the lung function of patients with CAD

**Fig. 6:**
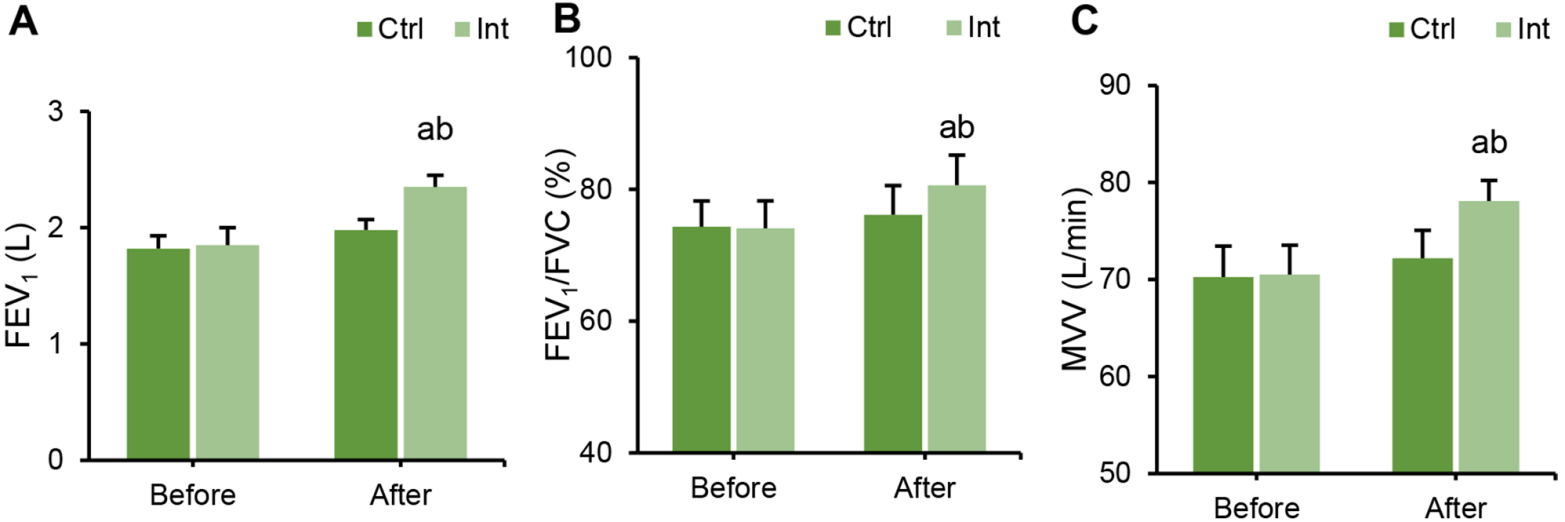
Lung function changes in the Ctrl group and Int group before and after intervention. (**A**) FEV_1_; (**B**) FEV_1_/FVC; (**C**) MVV; ^a^*P*<0.05 vs. that before intervention; ^b^*P*<0.05 vs. that of the postintervention Ctrl group

The differences in FEV_1_, FEV_1_/FVC, and MVV between groups before and after intervention were compared. Before intervention, there were no marked differences in FEV_1_, FEV_1_/FVC, or MVV between groups (*P* > 0.05). After intervention, FEV_1_, FEV_1_/FVC, and MVV indexes in the Int group were remarkably superior to those before intervention (*P* < 0.05), while the Ctrl group had no notable change (*P* > 0.05). After intervention, the FEV_1_, FEV_1_/FVC, and MVV indexes in the Int group were evidently higher than those in the Ctrl group (*P* < 0.05) (Fig. 6).

### Effect of RT on cardiopulmonary fitness and exercise ability of CAD patients

**Fig. 7:**
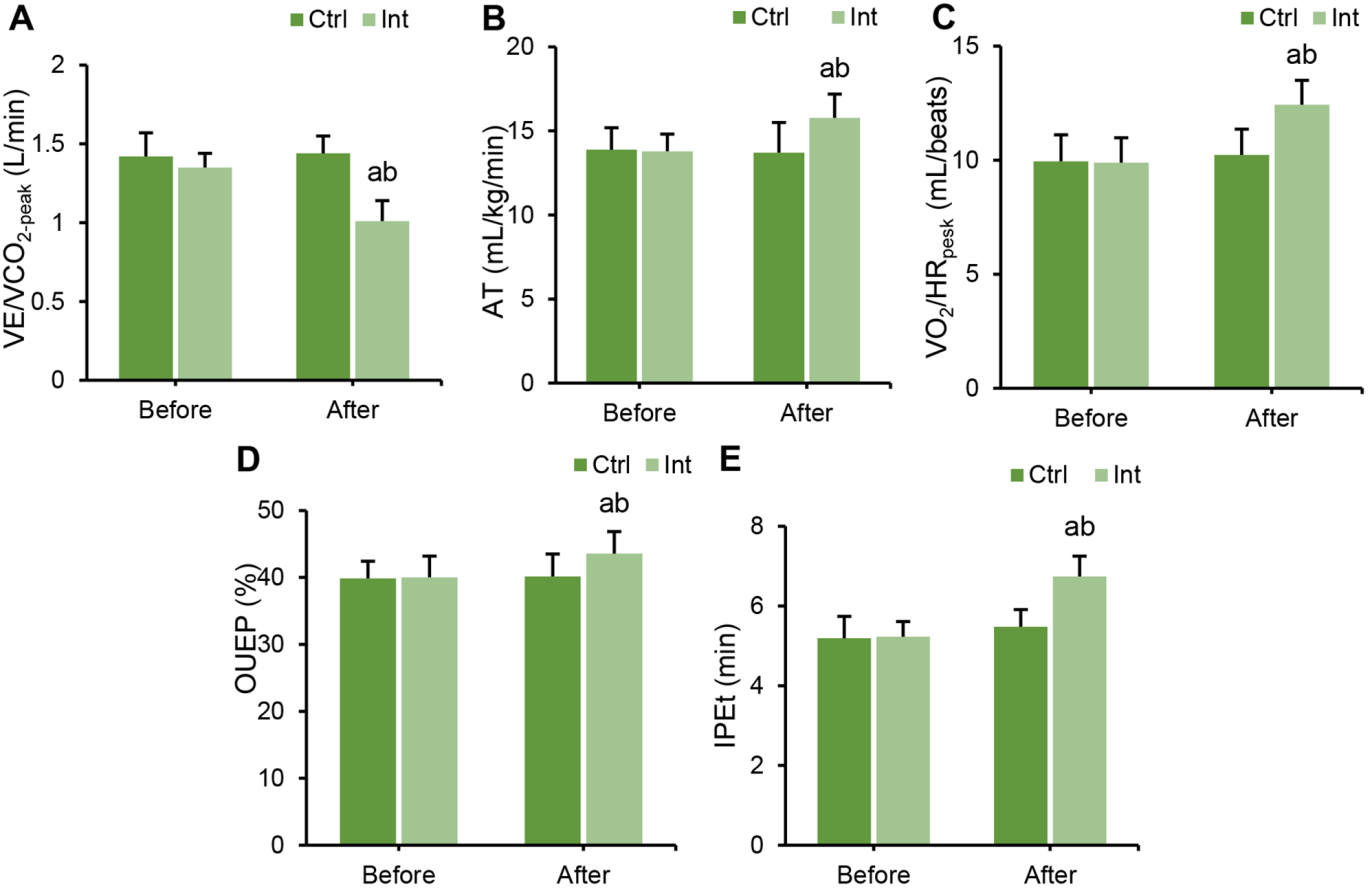
Cardiopulmonary fitness function was detected by CPET before and after intervention in the Ctrl group and Int group. (**A**) VE/VCO_2 peak_; (**B**) AT; (**C**) VO_2_/HR _peak_; (**D**) OUEP; (**E**) IPEt. ^a^*P*<0.05 vs. before intervention; ^b^*P*<0.05 vs. postintervention Ctrl group

CPET was performed, and the differences in cardiopulmonary fitness indexes (VO_2_-_peak_, AT, VO_2_/HR _peak_, OUEP and IPEt) between groups before and after intervention were compared. Before intervention, there were no marked differences in VE/VCO_2_-_peak_, AT, VO_2_/HR _peak_, OUEP, or IPEt between groups (*P* > 0.05). After intervention, the indexes of VE/VCO_2_-_peak_, AT, VO_2_/HR _peak_, OUEP, and IPEt in the Int group were evidently superior to those before intervention (*P* < 0.05) (Fig. 7).

**Fig. 8:**
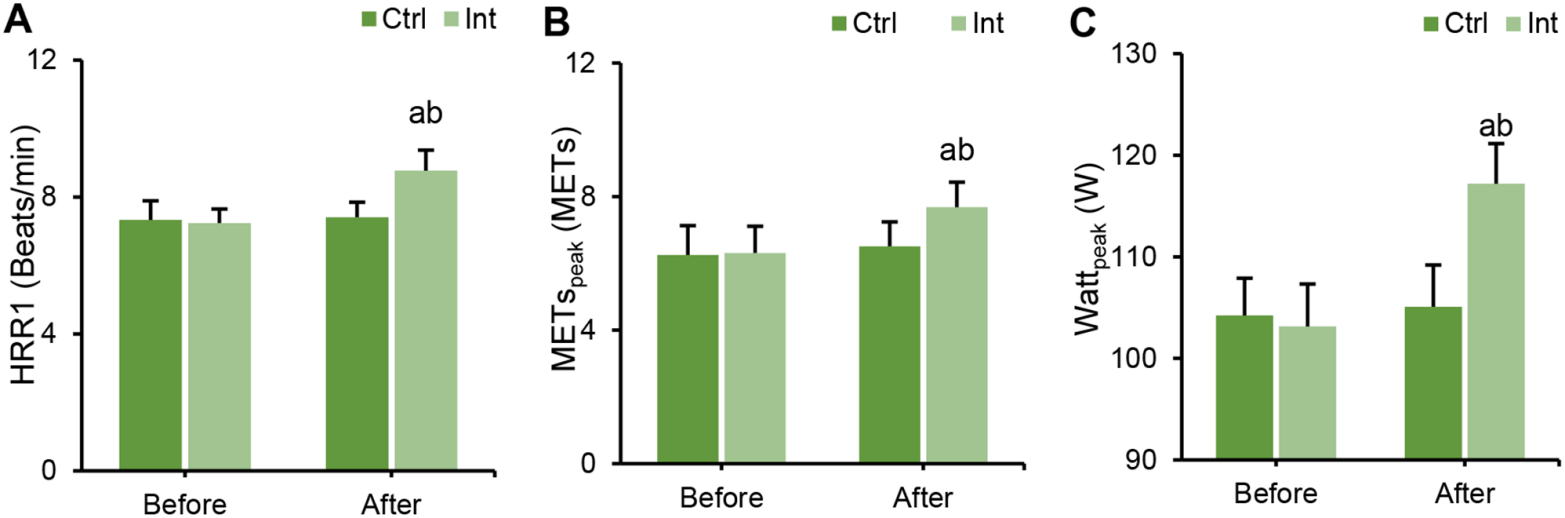
Changes in exercise ability measured by CPET in the Ctrl group and Int group before and after intervention. (**A**) HRR1; (**B**) MET _peak_; (**C**) Watt _peak_; ^a^*P*<0.05 vs. before intervention; ^b^*P*<0.05 vs. postintervention Ctrl group

The differences in HRR1, MET _peak_, and Watt _peak_ between groups before and after intervention were compared using CPET. Before intervention, there were no marked differences in HRR1, MET _peak_, or Watt _peak_ between groups (*P* > 0.05). After intervention, the indexes of HRR1, MET _peak_, and Watt _peak_ in the Int group were notably higher than those before intervention (*P* < 0.05), while the Ctrl group showed no notable change (*P* > 0.05). After intervention, the indexes of HRR1, MET _peak_, and Watt _peak_ in the Int group were evidently superior to those in the Ctrl group (*P* < 0.05) (Fig. 8).

### Effect of RT on anxiety and depression in CAD patients

**Fig. 9:**
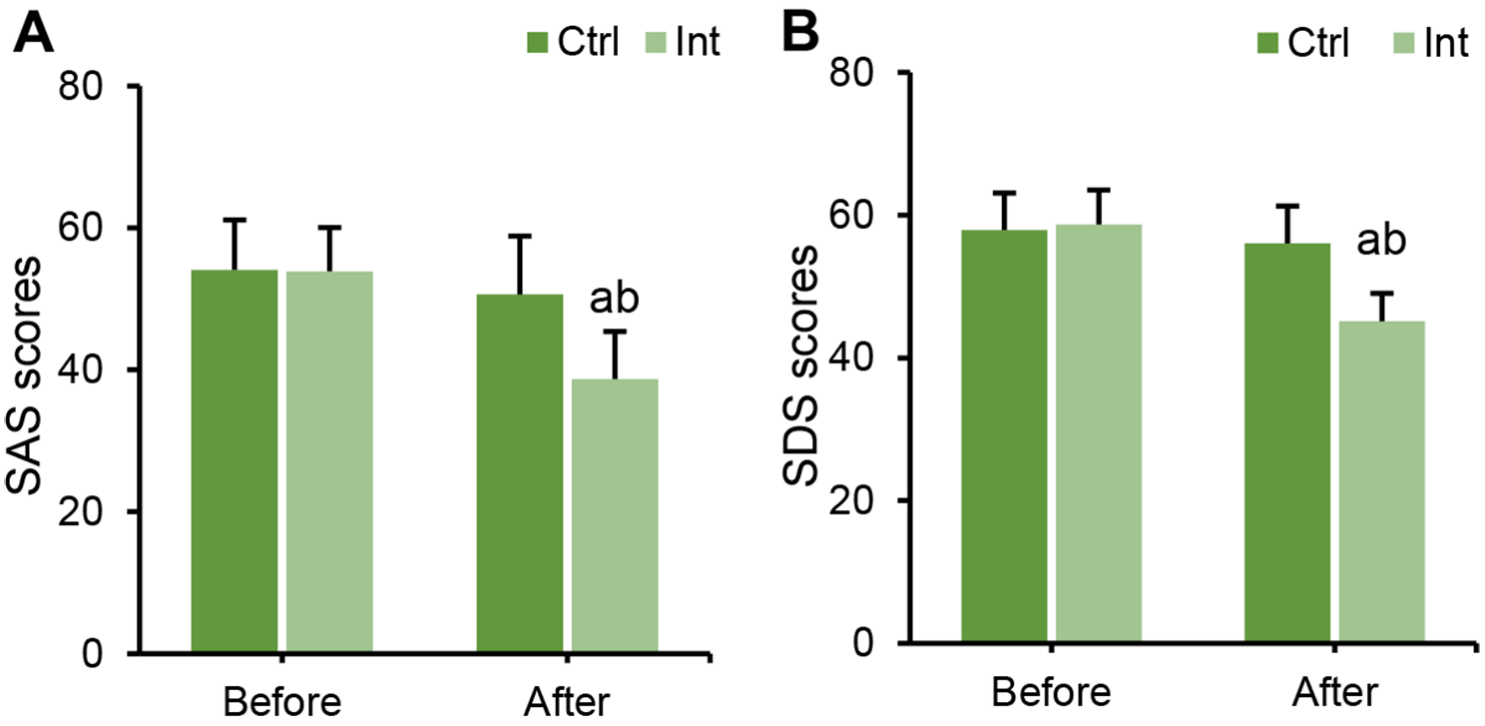
Changes in anxiety and depression before and after intervention in the Ctrl group and Int group. (**A**) SAS score of anxiety; (**B**) SDS score for depression; ^a^*P*<0.05 vs. that before intervention; ^b^*P*<0.05 vs. that of postintervention Ctrl group

SAS and SDS scale scores were compared between groups before and after intervention. Before intervention, there was no considerable difference in SAS and SDS scores between groups (*P* > 0.05). After intervention, the SAS and SDS scores of patients in the Int group were obviously inferior to those before intervention (*P* < 0.05), while the Ctrl group showed no notable change (*P* > 0.05). After intervention, SAS and SDS scale scores in the Int group were greatly inferior to those in the Ctrl group (*P* < 0.05) (Fig. 9).

### Effect of RT on the health status of CAD patients

**Fig. 10:**
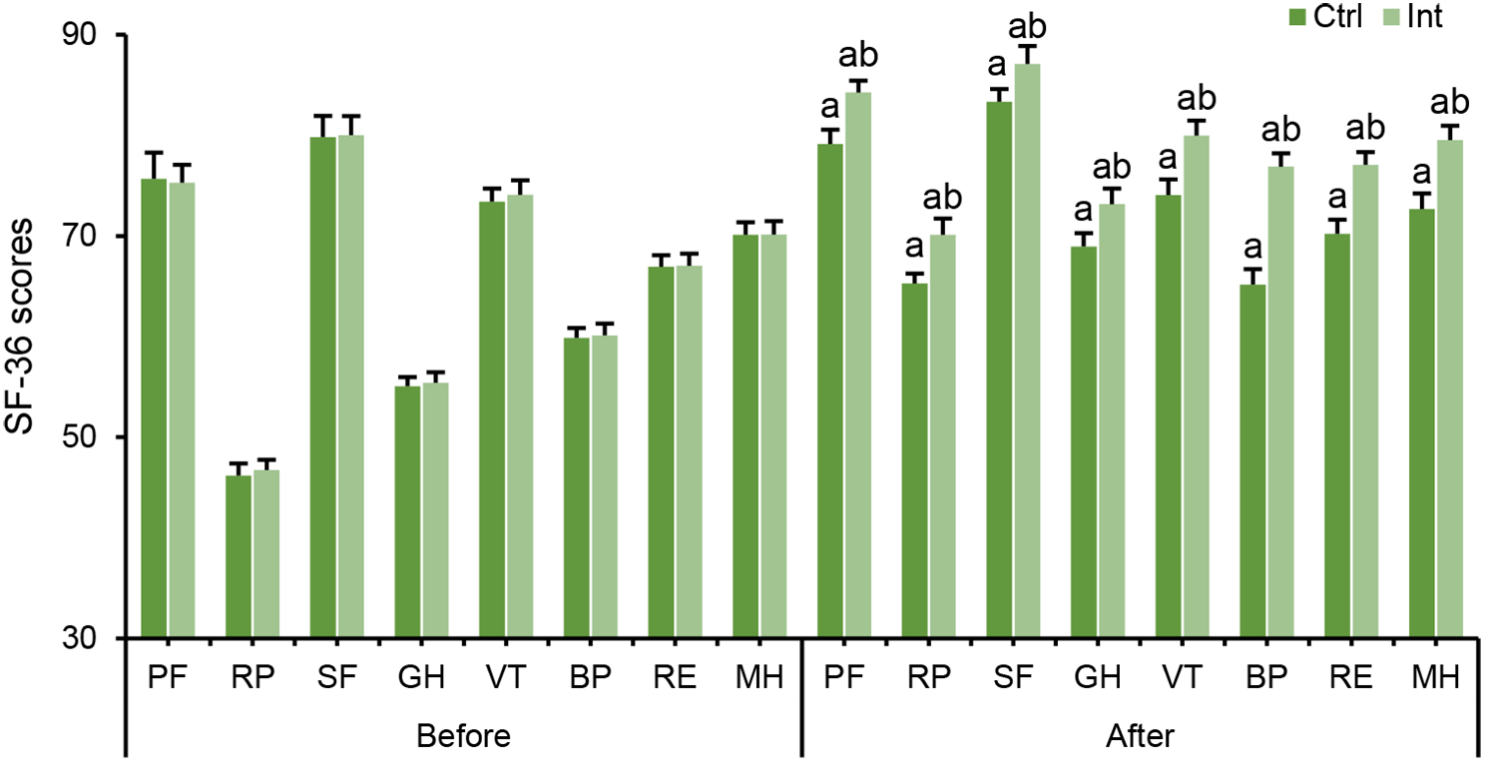
Changes in cardiac function in the Ctrl group and Int group before and after intervention. (**A**) LVEF; (**B**) LVEDD; (**C**) LVESD. ^a^*P*<0.05 vs. before intervention; ^b^*P*<0.05 vs. postintervention Ctrl group

The difference in SF-36 scores between groups before and after intervention was compared. Before intervention, there was no considerable difference in SF-36 scale scores between groups (*P* > 0.05). After intervention, SF-36 scale scores in the Ctrl group and Int group were drastically superior to those before intervention (*P* < 0.05). After intervention, the SF-36 score in the Int group was evidently superior to that in the Ctrl group (*P* < 0.05) (Fig. 10).

## Discussion

The cardiac rehabilitation of CAD patients is closely correlated with their quality of life and survival rates. Numerous studies have confirmed the significant benefits of cardiac rehabilitation for CAD patients, with ET being a crucial component that effectively enhances patients’ cardiopulmonary function [18–20]. In light of this, our study developed an aerobic exercise-based cardiac rehabilitation approach for patients with CAD who have undergone PCI. We investigated the impact of rehabilitation training on patients’ exercise capacity and cardiopulmonary function and assessed the changes in cardiopulmonary adaptability and exercise capacity in CAD patients after undergoing cardiac rehabilitation training.

First, the study analyzed the assessment of patients’ exercise capacity and cardiopulmonary function after rehabilitation training. The results indicated that in the Int group, postintervention, LVEDD and LVESD levels decreased, while LVEF, FEV1, FEV1/FVC, and MVV increased. These findings suggest that ET intervention has a positive impact on improving the cardiac and pulmonary function of patients. Consistent with related research, long-term aerobic exercise has been shown to significantly enhance cardiopulmonary function and exercise endurance in cardiovascular disease patients after PCI [21]. Moreover, long-term aerobic exercise can improve the morphological and structural aspects of the cardiovascular system, enhancing physiological adaptability [22]. Building upon these findings, the study further employed CPET to assess cardiopulmonary adaptability parameters, including Ve/VCO_2_-_peak_, AT, VO_2_/HR _peak_, OUEP, and IPEt. The study revealed that after the intervention, these parameters significantly improved in the Int group, indicating a positive impact of ET intervention on enhancing patients’ cardiopulmonary adaptability and exercise capacity. Sandercock et al. (2013) [23] also highlighted in their research that cardiac rehabilitation training can enhance patients’ cardiopulmonary function. Trachsel et al. (2021) employed CPET to assess changes in cardiac function in CAD patients after aerobic training and found that aerobic exercise can improve patients’ cardiopulmonary adaptability [24]. In CPET testing, the MET _peak_ can be used to evaluate patients’ exercise capacity and prognosis, with each additional 1 MET resulting in a 12% increase in the survival rate of CAD patients [25]. The higher the exercise capacity is, the lower the probability of cardiovascular events, such as myocardial ischemia, occurring in CAD patients. Moneghetti et al. (2017) [26] used imaging and CPET techniques to assess the end-systolic reserve of the left ventricle in patients with dilated cardiomyopathy and found a correlation between LVEF and VO_2_-_peak_. These results suggest that CPET parameters can, to a certain extent, reflect a patient’s LVEF and can be used to evaluate the cardiopulmonary functional status of CAD patients after PCI.

Furthermore, the study revealed that TC, TG, and LDL levels were significantly decreased in the Int group, while HDL levels were markedly increased (*P* < 0.05). Dyslipidemia is a major risk factor for CAD [27], indicating that aerobic exercise intervention has a positive impact on improving patients’ lipid profiles. This aligns with the findings of Batalik et al. (2022) [28]. The study also observed a significant decrease in HR and RPE, as well as an elevation in the ST segment in CAD patients following aerobic ET. This may be attributed to the fact that long-term aerobic exercise can lower heart rate, adjust the autonomic nervous system, and enhance ventricular volume expansion adaptation, thereby reducing cardiac workload and fatigue. A descending ST segment is a signal of myocardial ischemia, and the opposite is true [29]. RPE reflects the level of fatigue in physiological, psychological, and mental aspects and can indicate anxiety and depression levels in CAD patients [30]. This study also found that anxiety and depression levels in the Int group significantly decreased, and overall health status improved, indicating that aerobic training has a positive impact on enhancing patients’ mental health and overall quality of life.

In recent years, research on cardiac rehabilitation techniques has become increasingly prevalent. However, barriers to cardiac rehabilitation training for CAD patients persist due to issues related to patient engagement, inadequate rehabilitation resources, the financial burden of rehabilitation, and the influence of mental health concerns. Recent systematic reviews and meta-analyses have indicated that remote rehabilitation has a positive impact on cardiac rehabilitation and the treatment of CAD patients [31–33]. It can enhance patient engagement, personalized treatment, and psychological support. However, limitations remain due to the immaturity of technology, data privacy and security, medical oversight, and the effectiveness and safety of treatment. Consequently, future research should delve deeper into remote rehabilitation and mobile applications, personalized rehabilitation, and the effectiveness of mental health and rehabilitation.

## Conclusions

It was found that ET can improve cardiopulmonary function and exercise ability of CAD patients after PCI and can also improve patients’ anxiety and depression and improve their quality of life. However, there are still shortcomings in the study, such as limited sample size, no long-term follow-up observation of improvement effect, and no investigation into the occurrence of adverse reactions in patients during exercise. In conclusion, this study can provide a reference for the application and promotion of exercise rehabilitation in CAD patients.

## Data Availability

The datasets used and/or analyzed in the present study are available from the corresponding author upon reasonable request.
